# Analysis of Competitive Endogenous Mechanism and Survival Prognosis of Serum Exosomes in Ovarian Cancer Patients Based on Sequencing Technology and Bioinformatics

**DOI:** 10.3389/fgene.2022.850089

**Published:** 2022-06-30

**Authors:** Xia Li, Yurong Wang, Chunju Xu, Xirenguli Reheman, Yuxi Wang, Rong Xu, Jiahui Fan, Xueying Huang, Linna Long, Siying Yu, He Huang

**Affiliations:** ^1^ Department of Histology and Embryology, School of Basic Medical Sciences, Xinjiang Medical University, Urumqi, China; ^2^ Department of Gynecology, Affiliated Tumor Hospital of Xinjiang Medical University, Urumqi, China; ^3^ Department of Histology and Embryology, Xiangya School of Medicine, Central South University, Changsha, Hunan China

**Keywords:** ovarian cancer, serum exosomes, sequencing technology, bioanalysis, epigenetics, differential expression, target genes

## Abstract

**Background:** We determined the competitive endogenous in serum exosomes of ovarian cancer patients *via* sequencing technology and raw signal analysis. We performed an in-depth study of the potential mechanisms of ovarian cancer, predicted potential therapeutic targets and performed survival analysis of the potential targets.

**Methods:** Serum exosomes from three ovarian cancer patients were used as the experimental group, serum exosomes from three patients with uterine fibroids were used as the control group, and whole transcriptome analysis of serum exosomes was performed to identify differentially expressed long noncoding RNAs (lncRNAs) and mRNAs in ovarian cancer. The miRcode database and miRNA target gene prediction website were used to predict the target genes. Cytoscape software was used to generate a competing endogenous RNA (ceRNA) network of competitive endogenous mechanism of serum exosomes in ovarian cancer, and the R language was used for Gene Ontology (GO) and Kyoto Encyclopedia of Genes and Genomes (KEGG) enrichment analyses of the target genes. Finally, the TCGA website was used to download clinical and expression data related to ovarian cancer, and the common potential target genes obtained previously were analyzed for survival.

**Results:** A total of 117 differentially expressed lncRNAs as well as 513 differentially expressed mRNAs (*p* < 0.05, |log2 fold change (FC)|≥ 1.0) were obtained by combining sequencing data and raw signal analysis, and 841 predicted target genes were reciprocally mapped by combining the data from the miRcode database and miRNA target gene prediction website, resulting in 11 potential target genes related to ovarian cancer (FGFR3, BMPR1B, TRIM29, FBN2, PAPPA, CCDC58, IGSF3, FBXO10, GPAM, HOXA10, and LHFPL4). Survival analysis of the above 11 target genes revealed that the survival curve was statistically significant (*p* < 0.05) for HOXA10 but not for the other genes. Through enrichment analysis, we found that the above target genes were mainly involved in biological processes such as regulation of transmembrane receptor protein kinase activity, structural molecule activity with elasticity, transforming growth factor-activated receptor activity, and GABA receptor binding and were mainly enriched in signaling pathways regulating stem cell pluripotency, bladder cancer, glycerolipid metabolism, central carbon metabolism of cancer, and tyrosine stimulation to EGFR in signaling pathways such as resistance to enzyme inhibitors.

**Conclusions:** The serum exosomal DIO3OS-hsa-miR-27a-3p-HOXA10 competitive endogenous signaling axis affects ovarian cancer development and disease survival by targeting dysregulated transcriptional pathways in cancer.

## Introduction

Ovarian cancer is one of the three major gynecological malignancies that seriously affect women’s health, with the second highest incidence of 5.0/100,000 and mortality of 3.1/100,000 and the third highest morbidity after that of cancers of the uterine corpus and cervix (Siegel et al., 2020). Although major progress has recently been made in surgery and chemotherapy for ovarian cancer, the incidence and case fatality rate are still increasing yearly (Rocconi et al., 2020). Due to the insidious onset of ovarian cancer, approximately 70% of patients have advanced disease at the time of discovery, and this cancer is highly malignant with a poor prognosis. The 5-year survival rate of ovarian cancer patients is only 47% (Kandalaft et al., 2020). Current treatments for ovarian cancer mainly include tumor cytoreductive surgery and the chemotherapeutic drugs platinum and paclitaxel (Onda et al., 2020). The complete response rate of this standard treatment regimen in advanced ovarian cancer can reach 40%–60%. However, more than 90% of patients who relapse after 8 months and develop resistance to chemotherapeutic drugs eventually succumb to ovarian cancer (Coleman et al., 2019). Thus, the prognosis of ovarian cancer needs to be further improved, and identification of novel therapeutic targets and strategies is necessary. Competitive endogenous refers to heritable changes in gene expression and function that do not involve DNA sequence alterations and consists of certain regulatory mechanisms, such as DNA methylation, histone modification, and RNA editing (Tran et al., 2016). Research has shown that competitive endogenous plays an important role in the development of a variety of major diseases. In ovarian cancer, endometrial cancer, and cervical cancer, many studies have demonstrated the effects of genetic and competitive endogenous mechanism on tumor initiation and progression (Papakonstantinoua et al., 2020; Oliveira et al., 2021; Xie et al., 2021). Long noncoding RNAs (lncRNAs) are a class of RNAs with transcripts longer than 200 nt that play important regulatory roles in gene expression, development and diseases and are mainly localized in the nucleus to mediate competitive endogenous mechanism (Hosono et al., 2017). In this study, we aimed to explore the signaling pathways of competitive endogenous mechanism in serum exosomes from ovarian cancer patients and their potential therapeutic targets using whole transcriptome sequencing, transmission electron microscopy (TEM), and Sanger analysis.

## Materials and Methods

### Patients and Samples

Serum samples of ovarian cancer patients and patients with uterine fibroids in our hospital were collected, exosomes were extracted, and whole transcriptome sequencing was performed to detect differences in expression. The experimental group was ovarian cancer patients (the samples were preoperative serum samples of ovarian cancer patients), the postoperative histopathological results were malignant, and the control group was patients with uterine fibroids (the samples were preoperative serum samples) who had no previous ovarian disease. The inclusion criteria were as follows: 1) those who met the diagnostic criteria of ovarian cancer (Lokshin, 2012) and 2) those with a telephone follow-up. The exclusion criteria were 1) those with severe cardiac, hepatic, and renal dysfunction, 2) those with other malignant tumors or systemic infectious diseases, 3) those with other gynecological diseases, 4) those who withdrew from the study before completion, and 5) those with incomplete clinical data.

## Methods

### Extraction of Serum Exosomes by Ultracentrifugation

The serum was thawed in medium at 37°C for 30 min and centrifuged at 2000 × g and 4°C, and the supernatant was transferred to a new centrifuge tube and centrifuged again at 10,000 × g and 4°C for 45 min to remove larger vesicles. The supernatant was extracted and filtered through a 0.45 μM filter membrane, and the filter was collected. The filter was transferred to a new centrifuge tube, and an ultrarotor was selected and used at 4°C and 100,000 × g for 70 min. For removal of the supernatant, the supernatant was resuspended in 10 ml of prechilled 1 × PBS, and an ultrarotor was selected for centrifugation at 4°C and 100,000 × g and ultracentrifugation for 70 min. The supernatant was removed and resuspended in 100 μl of prechilled 1 × PBS. A total of 20 μl was collected for identification under electron microscopy, and the remaining exosomes were stored at −80°C.

### Transmission Electron Microscopy Observation

Ten microliters of the exosomes was removed, 10 μl of the sample was pipetted dropwise onto a copper grid to precipitate for 1 min, and filter paper was pipetted off the floating liquid. Uranium acetate (10 μl) was added dropwise onto the copper grid to precipitate the sample for 1 min, and filter paper was pipetted off the floating liquid. The samples were dried at room temperature for several minutes at 100 kV for electron microscopy imaging (Rikkert et al., 2019). TEM imaging results were obtained.

### Differential Expression Screening

The sequencing data were background corrected and normalized, and expression values were calculated using the Bioconductor R package in R. The DESeq2 package in R was used to calculate the differentially expressed lncRNAs and mRNAs between the two groups, with *p* < 0.05 and the magnitude of the expression change ≥ twofold [|log2 fold change (FC)| ≥ 1.0]. We screened the differentially expressed genes (DEGs), log2 FC ≥ 1.0 represents lncRNAs and mRNAs with upregulated expression, and log2FC ≤ −1.0 represents lncRNAs and mRNAs with downregulated expression. Finally, the differentially expressed lncRNAs (DElncRNAs) and mRNAs, that is, DElncRNAs as well as DEGs in the ovarian cancer and uterine leiomyoma control groups, were obtained. Heatmap plotting and cluster analysis of the screened DElncRNAs and DEGs were performed using the Heatmap package, and the *p* values in the data were −log10 transformed and −log10 (*p* values) grouped according to log2 FC (the upregulated lncRNA group, downregulated lncRNA group, and lncRNA group without statistical significance as well as the upregulated DEG group, downregulated DEG group, and DEG group without statistical significance). The post-treatment data were imported into GraphPad Prism 8 to generate volcano plots.

### MicroRNAs (miRNAs) Predicted to Bind to lncRNAs

The sequencing datasets were differentially analyzed using the miRcode database, and the resulting DElncRNAs were predicted to be bound by miRNAs to further explore the underlying pathogenesis of the disease. The processed data were compared with the miRcode database for the DElncRNAs with upregulated and downregulated expression according to the set filtering criteria (http://www.mircode.org). Among the “Highly conserved microRNA families” dataset, miRNAs with potential binding to DElncRNAs were comparatively analyzed.

### Prediction of miRNA Target Genes

With the miRNAs derived from the previous step that were potentially bound by DElncRNAs, miRNA target gene prediction was performed. The miRNAs that potentially bound to the DElncRNAs were separately input into the online miRNA target gene prediction websites miRDB, miRTarBase, and TargetScan for target gene prediction, and the resulting predicted target genes were aligned and mapped with the DEGs obtained from sequencing. The differentially expressed lncRNAs—miRNA—mRNAs were further obtained by Cytoscape 3.7.2 with the competing endogenous RNA (ceRNA) interaction network (Shannon et al., 2003).

### Gene Ontology and Kyoto Encyclopedia of Genes and Genomes Enrichment Analysis

The common potential target genes predicted from the previous step were entered into the DAVID database with species set as human (*Homo sapiens*) for Gene Ontology (GO) and Kyoto Encyclopedia of Genes and Genomes (KEGG) signaling pathway analyses (Huang et al., 2009a; Huang et al., 2009b). The GO analysis included the cellular component (CC), molecular function (MF) and biological process (BP) categories of the DEGs. Target genes were screened at *p* < 0.05 to identify the biological processes of potential target genes and major signaling pathways. Pathway diagrams were established using Bioconductor-Pathview in R software (version R x64 3.5.1).

### Survival Analysis of Potential Target Genes

The resulting common potential target genes from 1.2.5 were subjected to survival analysis using human clinical and expression data from TCGA (https://portal.gdc.cancer.gov/). R software was utilized to collate summary clinical data and further derive its expression matrix. Finally, the survival package was utilized to perform survival analysis on the common potential target genes obtained in the previous step.

## Results

### Characteristics of Serum Exosomes

**FIGURE 1:**
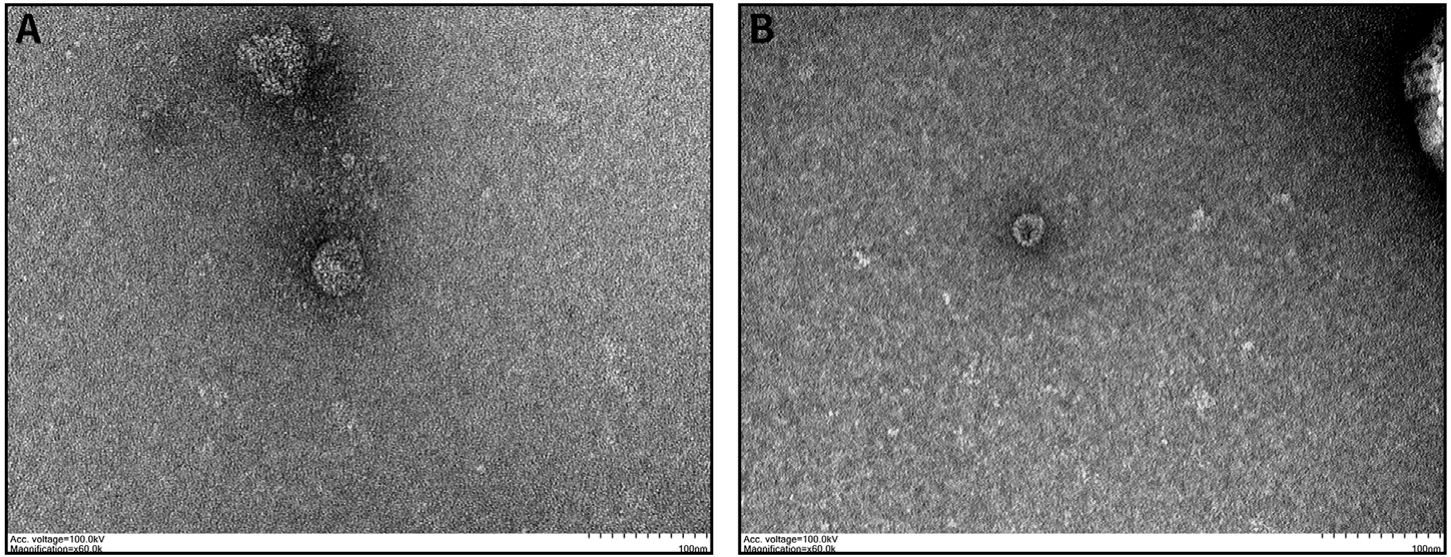
Characteristics of exosomes isolated from serum samples: **(A,B)** Serum-derived exosome morphology visualized by TEM, indicating that the diameter of the isolated exosomes was in the range of 30–200 nm.

To determine whether the particles isolated from serum were exosomes, we characterized the vesicles by TEM, size assessment, and analysis of protein quality. TEM images showed that the serum exosomes were morphologically intact, spherical, and uniform in size, with diameters ranging from 30 to 200 nm, which corresponds to the conventional size range of exosomes (Figure 1). As expected, the results of protein quantity assessment showed that the commonly used exosomal markers, such as CD9, CD63, CD81, and TSG101, were abundantly expressed in the isolated pellets, and all the above results confirmed the successful isolation of exosomes from serum samples.

### Screening of Differentially Expressed Genes

**FIGURE 2:**
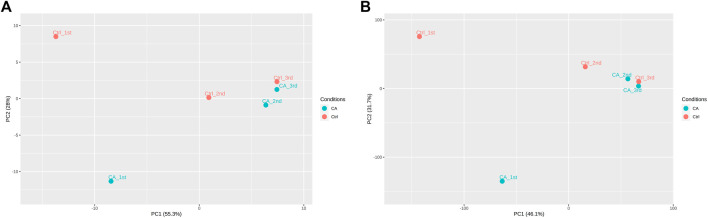
PCA distribution of ovarian cancer sequencing data: **(A)** PCA distribution of lncRNA data. **(B)** PCA distribution of mRNA data.

**FIGURE 3:**
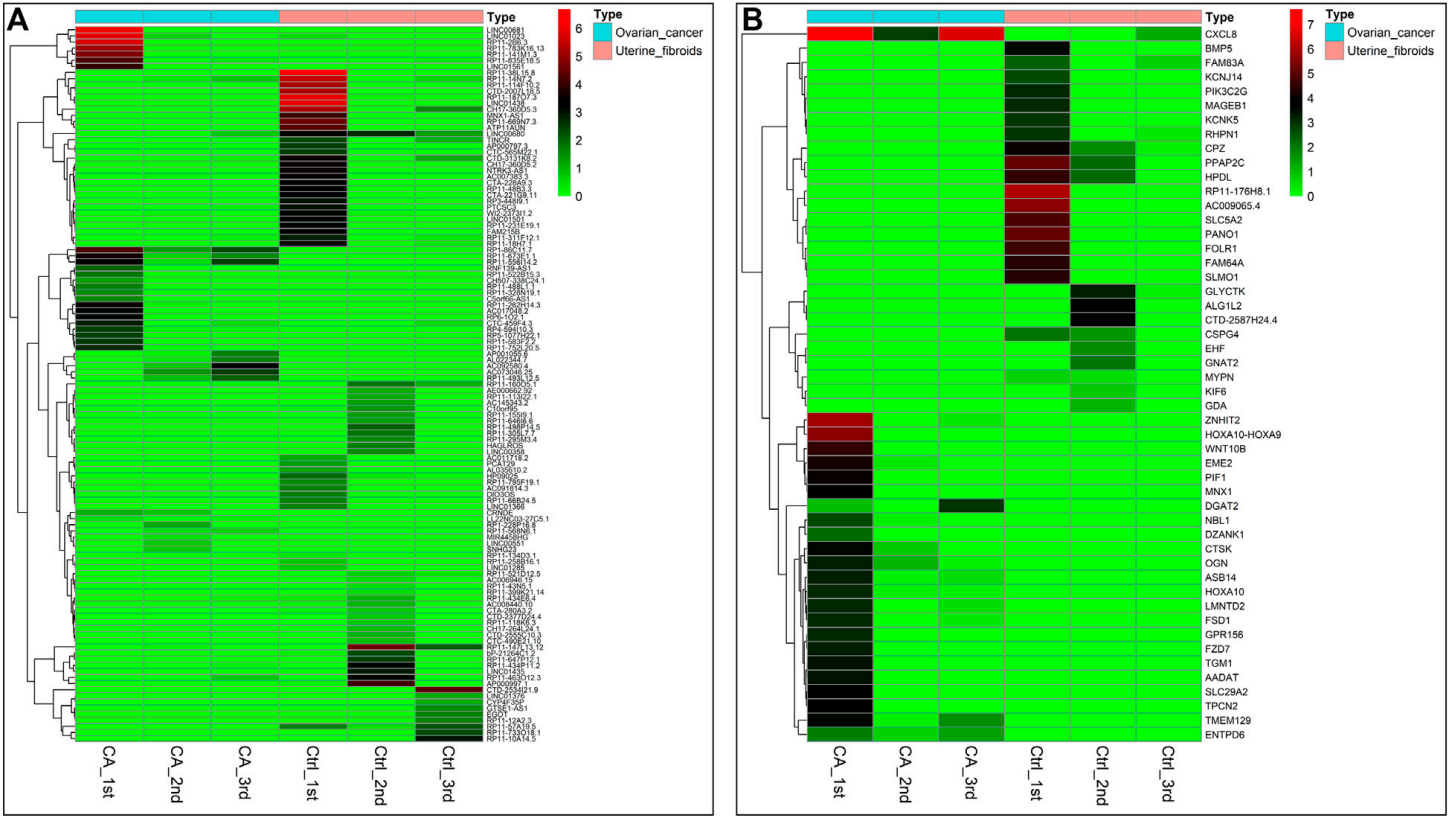
Heatmap of DEGs in ovarian cancer: **(A)** Heatmap of 117 DElncRNA clusters. **(B)** Top 50 most significant DEGs. Note: tissue samples are presented as columns; individual genes are represented as rows. In patients with ovarian cancer, red indicates genes with upregulated expression, and green indicates genes with downregulated expression. Top, blue represents the ovarian cancer group, and pink represents the uterine fibroid group.

**FIGURE 4:**
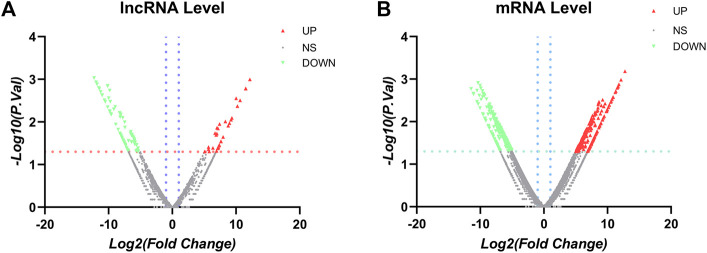
Volcano plots: **(A)** LncRNA volcano plots. **(B)** mRNA volcano plots. Note: the vertical blue line corresponds to the increase and of log_2_FC, while the horizontal orange line indicates *p* value <0.05. Red dots represent upregulated and statistically significant DElncRNAs, and green dots represent downregulated and statistically significant DElncRNAs. FC > 2.0 and *p* < 0.05 were used as standards, FC was log2 transformed, and the *p* value was −log10 transformed.

After establishment of screening conditions, we obtained three preoperative serum samples from ovarian cancer patients (aged 39.0 ± 6.0 years) and three serum samples from patients with uterine fibroids (aged 58.0 ± 13.0 years) collected in our hospital, and the baseline characteristics of the samples are shown in Table 1. The sequencing data were background corrected, normalized, and normalized using the R package, and the principal component analysis (PCA) of the corrected data distribution is shown in Figure 2. We used *p* < 0.05 and a ≥ twofold change in expression (|log_2_ FC| ≥ 1.0) as the criteria for the differentially expressed lncRNAs and coding RNAs, and 117 DElncRNAs—36 lncRNAs with upregulated expression and 81 lncRNAs with downregulated expression—and 513 differentially expressed mRNAs—231 mRNAs with upregulated expression and 282 mRNAs with downregulated expression—were selected in the sequencing data according to the *p* value. The top 50 significantly DElncRNAs and coding RNAs were screened and plotted as a heatmap (see Figure 3). Red represents upregulation of gene expression, and green represents downregulation of gene expression. The *p* value in the sequencing data after differential analysis was −log10 transformed, and −log10 (*p* value) was grouped according to log_2_ FC (the upregulated lncRNA group, downregulated lncRNA group, and lncRNA group without significant difference as well as the upregulated DEG group, downregulated DEG group, and DEG group without statistical significance). The post-treatment data were imported into GraphPad Prism 8 to generate a volcano plot (see Figure 4).

**TABLE 1:** Table of sample baseline characteristics.

Sample size	Age (year, ‾X ± SD)	Tumor stage			Sample
		I	II	III	
hysteromyoma (*n* = 3)	39.0 ± 6.0	—	—	—	Serum exosomes
Ovarian cancer (*n* = 3)	58.0 ± 13.0	IC(1)	—	IIIC(2)	Serum exosomes

### Prediction of lncRNA-Bound miRNAs

Analysis of sequencing data using the miRcode database was used to obtain DElncRNAs and the predicted binding miRNAs to further explore the underlying pathogenesis of the disease. The processed data were compared with the miRcode database according to the set filtering criteria for DElncRNAs. The DElncRNAs with up- and downregulated expression were (http://www.mircode.org/) in the “highly conserved microRNA families” dataset and were comparatively analyzed to obtain the miRNAs potentially bound to the DElncRNAs. The results are shown in Table 2.

**TABLE 2:** MiRNAs potentially bound by DElncRNAs.

lncRNA	miRNA
C10orf95	hsa-miR-503、hsa-miR-7、hsa-miR-7ab、hsa-miR-143、hsa-miR-1721、hsa-miR-4770、hsa-miR-150...
LINC00358	hsa-miR-141、hsa-miR-200a、hsa-miR-150、hsa-miR-5127、hsa-miR-1ab、hsa-miR-206...
FAM215B	hsa-miR-503、hsa-miR-139-5p、hsa-miR-205、hsa-miR-205ab、hsa-miR-217、hsa-miR-218...
EGOT	hsa-miR-135ab、hsa-miR-135a-5p、hsa-miR-141、hsa-miR-200a、hsa-miR-143、hsa-miR-1721...
CRNDE	hsa-miR-9、hsa-miR-9ab、hsa-miR-135ab、hsa-miR-135a-5p、hsa-miR-140、hsa-miR-140-5p...

### CeRNA, Network Construction

**FIGURE 5:**
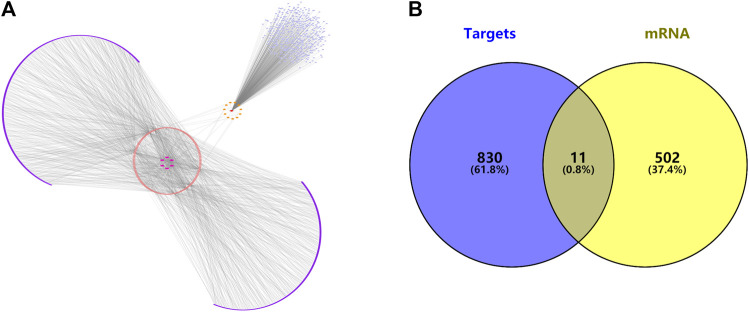
**(A)** The ceRNA interaction network of lncRNAs—miRNAs—mRNAs. **(B)** Venn diagram of predicted target genes and disease targets.

Using the miRNAs potentially bound to the DElncRNAs from the previous step, we performed miRNA target gene prediction and compared the data with the DEGs derived from the previous differential expression analysis (see Figure 5) to further explore the underlying pathogenesis of the disease. The miRNAs potentially bound to the DElncRNAs were input into the online miRNA target gene prediction websites miRDB, miRTarBase, and TargetScan for target prediction. The results are shown in Table 3, and Cytoscape 3.7.2 software was used to obtain the ceRNA interaction network of differentially expressed lncRNAs—miRNA—mRNA (see Figure 5B).

**TABLE 3:** MiRNA target gene prediction.

miRNA	Gene	miRDB	miRTarBase	TargetScan	Sum
hsa-miR-129-5p	SORBS2	1	1	1	3
hsa-miR-125b-5p	PPAT	1	1	1	3
hsa-miR-23b-3p	PTK2B	1	1	1	3
hsa-miR-129-5p	RSBN1	1	1	1	3
hsa-miR-135a-5p	STAT6	1	1	1	3
hsa-miR-24-3p	PER2	1	1	1	3
hsa-miR-1297	FRAT2	1	1	1	3
hsa-miR-10a-5p	CHL1	1	1	1	3
hsa-miR-107	LATS2	1	1	1	3
hsa-miR-24-3p	AVL9	1	1	1	3
...	...	—	—	—	—

### Survival Analysis of the Target Genes

**FIGURE 6:**
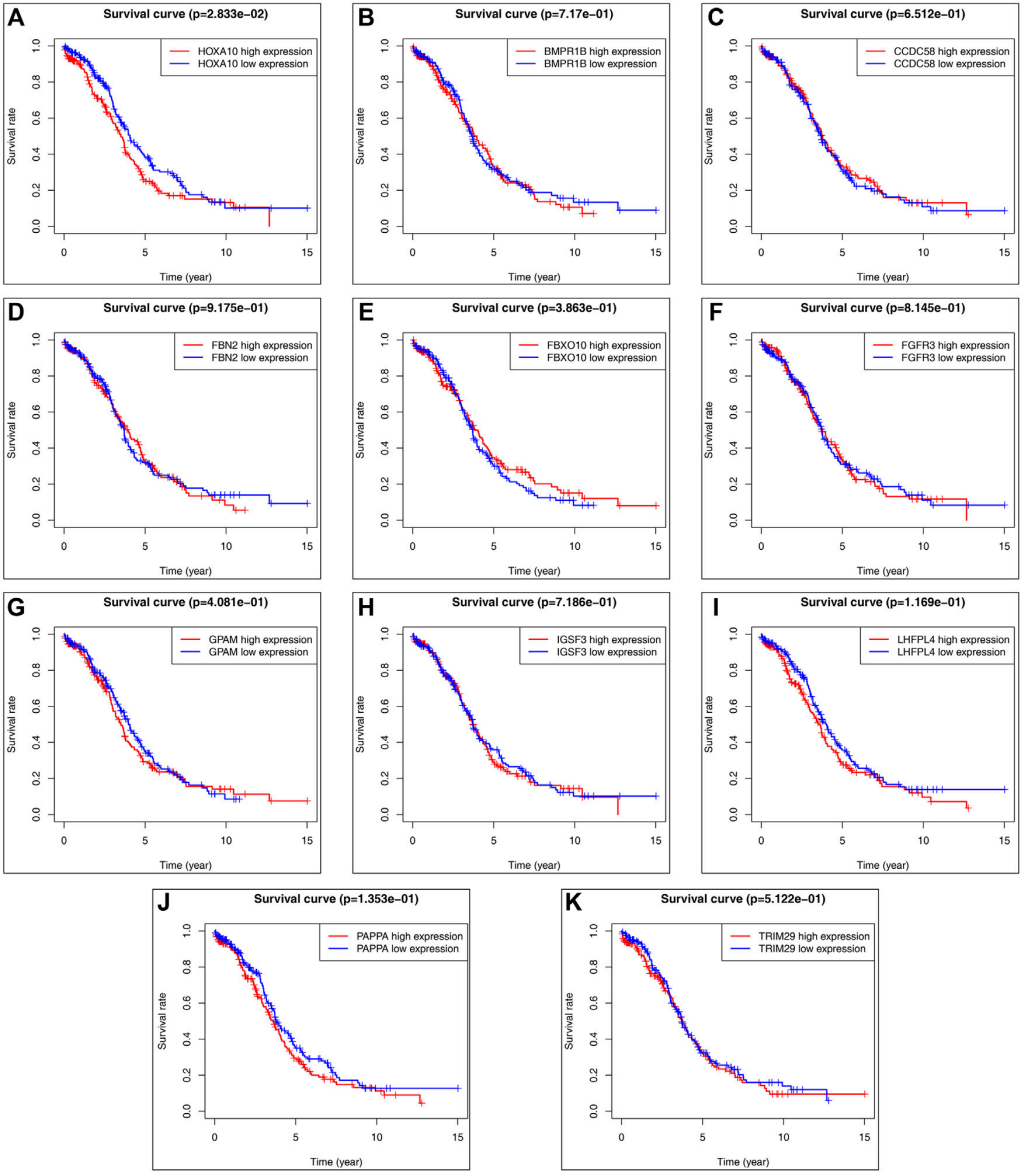
Predicted target gene survival curve: **(A)** HOXA10 gene survival curve. **(B)** BMPR1B gene survival curve. **(C)** CCDC58 gene survival curve. **(D)** FBN2 gene survival curve. **(E)** FBXO10 gene survival curve. **(F)** FGFR3 gene survival curve. **(G)** GPAM gene survival curve. **(H)** IGSF3 gene survival curve. **(I)** LHFPL4 gene survival curve. **(J)** PAPPA gene survival curve. **(K)** TRIM29 gene survival curve. (The red curve indicates high gene expression, the blue curve indicates low gene espression).

Utilizing TCGA (https://portal.gdc.cancer.gov/), we downloaded clinical and transcriptomic expression data related to ovarian cancer, 379 related datasets were generated according to the screening conditions set in the previous period, the pooled summary clinical data were collated using R software, and the expression matrix was further derived. Finally, the common potential target genes obtained in the previous period were subjected to survival analysis using the survival package, which revealed that the survival curve of the HOXA10 gene had statistical significance (*p* < 0.05), but none of the other indexes showed statistical significance (see Figure 6).

### Gene Ontology and Kyoto Encyclopedia of Genes and Genomes Pathway Enrichment Analysis of Target Genes

**FIGURE 7:**
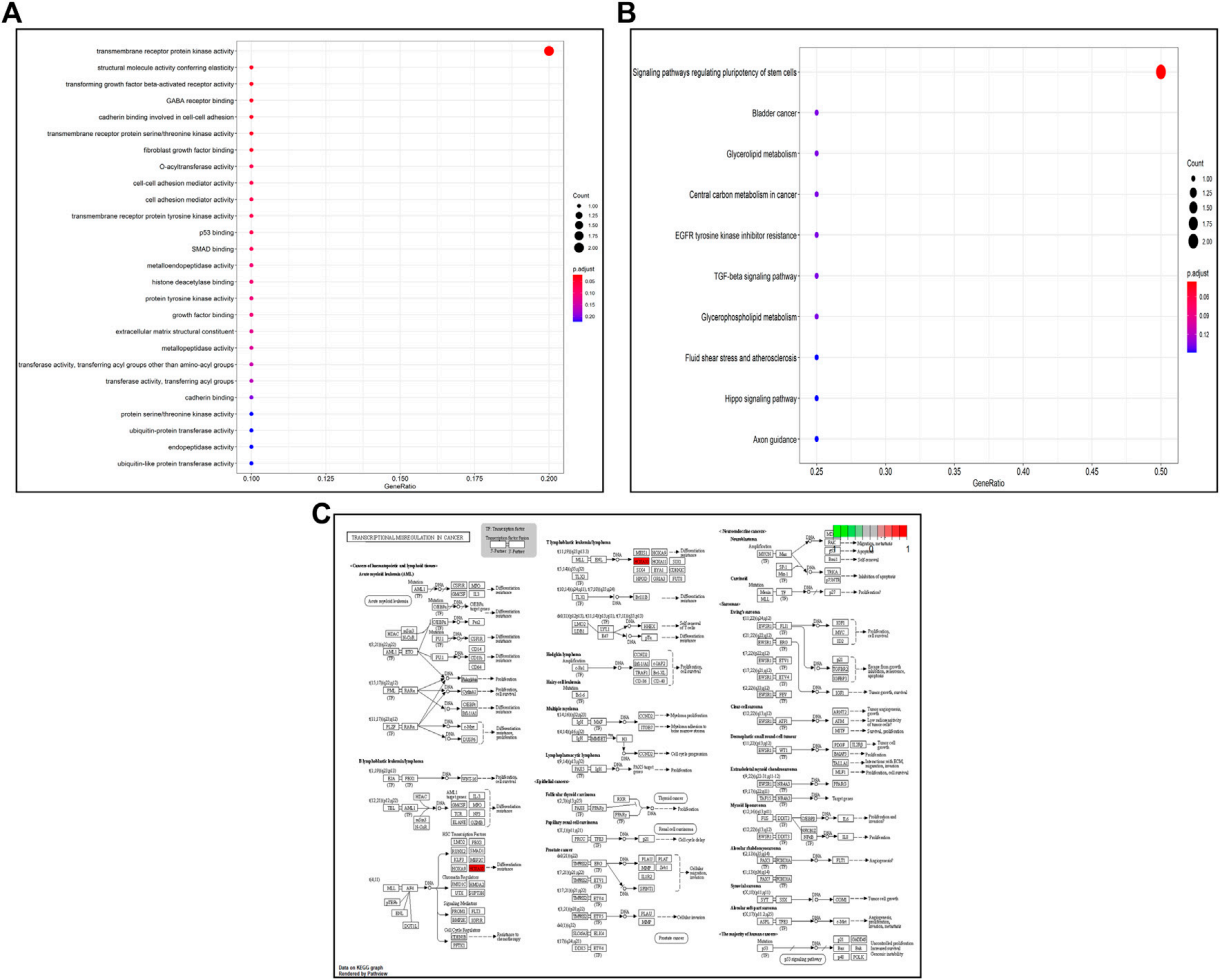
GO/KEGG enrichment analysis: **(A)** GO enrichment bubble plot of potential target genes. **(B)** KEGG enrichment bubble plot of potential target genes. **(C)** Transcriptional dysregulation in cancer signaling pathways.

GO and KEGG pathway enrichment analyses of potential target genes were performed using the DAVID database, and the GO functions of the core genes were mainly involved in transmembrane receptor protein kinase activity, structural molecule activity with elasticity, transforming growth factor-β activated receptor activity, and GABA receptor binding, as shown in Figure 7. The results of KEGG pathway enrichment analysis showed that the KEGG pathways were mainly involved in signaling pathways regulating stem cell pluripotency, bladder cancer, glycerolipid metabolism, central carbon metabolism in cancer, and resistance to EGFR tyrosine kinase inhibitors (see Figure 7B). A schematic diagram of the signaling pathways related to the HOXA10 gene is shown in Figure 7C.

## Discussion

Epigenetics refers to heritable changes in gene expression and function that arise through certain mechanisms that do not involve DNA sequence alterations, including regulatory mechanisms such as DNA methylation, histone modifications, and RNA editing (Portela and Esteller, 2010). Previous studies have shown that competitive endogenous plays an important role in the development of various major diseases (Navarro et al., 2014; Sarkargar et al., 2021; Szukiewicz et al., 2021). Many studies have demonstrated the influence of genetic and epigenetic modifications on tumor initiation and progression in ovarian cancer, endometrial cancer, and cervical cancer. In contrast to gene mutations, competitive endogenous do not act by altering the genomic sequence but by methylating modifications, histone modifications, miRNA regulation, etc. Aberrant methylation, histone modification errors, and miRNA dysregulation are closely associated with tumor cell proliferation, autophagy, apoptosis, cell-cell adhesion, invasion, and metastasis (Herceg and Vaissiere, 2011). Therefore, this study aimed to identify the DEGs in ovarian cancer patients with whole transcriptome sequencing technology and Sanger analysis to explore novel therapeutic targets and diagnostic strategies.

According to statistics, gene mutations account for up to 1/4 of ovarian cancer cases. BRCA1 and BRCA2 were shown to be susceptibility genes for ovarian cancer (Wu et al., 2017), and the BRIP1, RAD51C, rad51D and mismatch repair genes also play a role in this disease (Suszynska et al., 2020). The lncRNA HAND2-AS1/miR-340-5p/BCL2L11 axis was shown to promote proliferation and apoptosis of ovarian cancer through the ceRNA mechanism and affect patient survival (Chen et al., 2019). In addition, lncRNA MALAT can reach human umbilical vein endothelial cells in a paracrine manner *via* exosomes from the serum of ovarian cancer patients to regulate angiogenesis by altering the expression of angiogenesis-related genes (Qiu et al., 2018). Finally, the lncRNA LINC00161/miR-128/MAPK pathway was shown to promote the development of platinum resistance in ovarian cancer tissues (Xu et al., 2019). In conclusion, our results demonstrated that aberrant expression of lncRNAs can affect several processes, such as tumor proliferation, invasion, metastasis, epithelial mesenchymal transition, vascularization, platinum chemoresistance, and regulation of ovarian cancer occurrence and development. Studies on the mechanism of lncRNAs in ovarian cancer have mainly focused on competitive endogenous: lncRNAs and miRNAs interact with each other, lncRNAs can act as sponges for miRNAs, and changes in their expression can lead to changes in miRNA expression, which in turn causes abnormal expression of mRNAs. In addition, lncRNAs have a relationship with serum exosomes; thus, we extracted serum exosomes, identified the DEGs in ovarian cancer patients, investigated the interrelationships between lncRNAs and miRNAs and between mRNAs and exosomes, and actively searched for specific serum biological markers in ovarian cancer patients to improve the early diagnosis of ovarian cancer.

In this study, we performed whole transcriptome sequencing to identify 117 DElncRNAs as well as 513 differentially expressed mRNAs by extracting serum exosomes from ovarian cancer patients and combining 841 predicted target genes derived from the miRcode database and miRNA target gene prediction website to obtain 11 potential target genes related to ovarian cancer (FGFR3, BMPR1B, TRIM29, FBN2, PAPPA, CCDC58, IGSF3, FBXO10, GPAM, HOXA10, and LHFPL4). Moreover, GO/KEGG enrichment analysis of the above 11 target genes revealed that these targets were mainly involved in regulating biological processes such as transmembrane receptor protein kinase activity, structural molecule activity with elasticity, transforming growth factor-activated receptor activity, and GABA receptor binding and were mainly enriched in signaling pathways regulating stem cell pluripotency, bladder cancer, glycerolipid metabolism, cancer hub carbon metabolism, resistance to EGFR tyrosine kinase inhibitors and other signaling pathways. Finally, survival analysis of the above targets identified a statistically significant (*p* < 0.05) survival curve only for the HOXA10 gene, which is mainly involved in the DIO3OS-hsa-miR-27a-3p-HOXA10 competitive endogenous signaling axis that affects the occurrence and development of ovarian cancer and disease survival.

The lncRNA DIO3OS has been implicated in the development and progression of various tumors (Cui et al., 2019; Wang et al., 2020; Wang et al., 2021); however, its specific role in the development and progression of ovarian cancer has not been investigated. In addition, hsa-miR-27a-3p has been shown to affect tumor proliferation, invasion, metastasis in glioblastoma, intrahepatic cholangiocarcinoma, and other malignancies (Xu et al., 2020; Salmani et al., 2021). Moreover, upregulated expression of HOXA10 was shown to promote epithelial mesenchymal transition as well as proliferation, migration and invasion of ovarian cancer cells and decrease patient survival (Jiang et al., 2014; Liu et al., 2018; Nie et al., 2021). Therefore, the target gene HOXA10 may affect the prognosis of patients with ovarian cancer by regulating dysregulated transcriptional pathways in cancer while affecting processes such as tumor proliferation, invasion, metastasis, epithelial mesenchymal transition, vascularization, and platinum chemoresistance.

## Conclusion

In conclusion, the serum exosomal DIO3OS-hsa-miR-27a-3p-HOXA10 competitive endogenous mechanism signaling axis affects ovarian cancer development and disease survival by targeting dysregulated transcriptional pathways in cancer.

## Data Availability

The original contributions presented in the study are publicly available. This data can be found here: GSE194238.
